
JOURNAL INFORMATION
==============================
NLM Title Abbreviation: Hist Cienc Saude Manguinhos
Journal ID: Hist Cienc Saude Manguinhos
NLM Journal ID: 9513999
Journal ID: hcsm

História, Ciências, Saúde - Manguinhos

ISSN: 0104-5970
EISSN: 1678-4758
Publisher: Casa de Oswaldo Cruz - Fiocruz

ARTICLE INFORMATION
==============================
PMCID: PMC10425154
DOI: 10.1590/S0104-59702023000100038
Article ID: 01509
Article version: 1
Subjects: Resenhas

Rural sociology towards the third way: from the United States to Brazil

A sociologia rural em direção à terceira via: dos Estados Unidos ao Brasil

http://orcid.org/0000-0002-6460-495X Mesquita Gustavo i
i Professor, Universidade de Brasília Brasília – DF – Brasil gustavormesquita@gmail.com
Electronic publication date: 2023 Aug 11
Publication date: 2023
Volume: 30
Electronic Location ID: e2023038
Product: LOPES Thiago da Costa  
Em busca da comunidade: ciências sociais, desenvolvimento rural e diplomacia cultural nas relações Brasil-EUA (1930-1950).
Rio de Janeiro: Editora Fiocruz. 2020. 281.
License: This is an Open Access article distributed under the terms of the Creative Commons Attribution License, which permits unrestricted use, distribution, and reproduction in any medium, provided the original work is properly cited.
License URL: https://creativecommons.org/licenses/by/4.0/

Figure count: 0
Table count: 0
Equation count: 0
Reference count: 6

==============================

Foi com satisfação e surpresa que li o livro de Thiago da Costa Lopes. Deixou-me num verdadeiro estado reflexivo, ao final da leitura. Sobre as reflexões provocadas, quero dizer primeiro que vejo esse como um daqueles poucos livros em que o autor consegue pôr em prática o que se ensina em teoria, mas geralmente fica limitado à própria teoria ensinada. Refiro-me ao projeto de fazer áreas do conhecimento conversarem, no qual Bloch (2001) continua sendo inspiração teórica das mais estimulantes. Para nos contar uma história da sociologia rural, Lopes faz amplas, bastante amplas análises do pensamento que se definiu ao redor do comunitarismo. Ao mesmo tempo, reconstrói a história da disciplina acadêmica que expressou tal pensamento na forma de uma sociologia rural. Tudo indica que a chamada religação filosófica das humanidades, ou mais modestamente a conjugação entre história e ciências sociais, é encarada de frente pelo autor como algo planejado. O leitor mais curioso logo vê essa conjugação sendo realizada cuidadosa e pacientemente na condução interdisciplinar da pesquisa e na costura do texto sem fronteiras do conhecimento, pleno de diálogos disciplinares, do início ao fim.

Digo que as análises de Lopes sobre o comunitarismo têm amplitude por mais de um motivo. A começar pela abordagem desenvolvida, o autor estuda a formação desse pensamento, o que ele chama de utopia comunitária, em consonância com a dimensão transnacional da circulação de textos, ideias e intelectuais importantes para a utopia pelo menos entre as décadas de 1930 e 1950. E tal abordagem se faz presente no estudo não porque é a última moda historiográfica, se assim quisermos chamar o que João Marcelo Ehlert Maia (2017) aponta como sendo tendências metodológicas recentes, às vezes se passando por moda intelectual ou superestimadas como grande novidade, na pesquisa de história da sociologia e de pensamento social brasileiro.

Mas, poder-se-ia dizer jocosamente, "comigo não, violão, comigo não", porque modas não parecem ser o que anima Lopes em sua investigação. Ocorre que o olhar transnacional realmente o ajuda a ir além e a entender o que estava por trás de teses sociológicas acerca do desenvolvimento rural. Ele faz um longo e detalhado percurso investigativo em que vai puxando e revelando os fios que ligaram as ideias do sociólogo brasileiro José Arthur Rios (1921-2017) ao centro global do comunitarismo de terceira via: a sociologia rural dos Estados Unidos e suas políticas públicas implementadas no *New Deal* de Roosevelt. Também revela as trocas entre professores que consolidaram a sociologia rural nos Estados Unidos e professores que tentaram o mesmo feito no Brasil. T. Lynn Smith (1903-1976) e José Arthur Rios estão no cerne dessa narrativa.

É todo um circuito acadêmico de livros de referência, conceitos, métodos, técnicas e teorias de intervenção social e reforma agrária que Lopes traz à tona nessa espécie de arqueologia da sociologia rural de Smith e Rios. Nisso, acaba aproximando-se da noção de história do conhecimento de Foucault (2008), ainda que com ele não dialogue explicitamente. Lopes demonstra domínio tanto da sociologia rural quanto da história global do comunitarismo. Pratica bem a arte da escrita da história com seu estilo elegante, de modo a fazer com que o leitor percorra, sem sofrimento, amplos e especializados debates entre sociólogos, dirigentes políticos e diplomatas sobre problemas agrários do mundo e particularmente dos Estados Unidos e do Brasil. Sua capacidade investigativa nos leva a compreender como os debates amadureceram nos anos iniciais da Guerra Fria, ao longo dos quais o antitotalitarismo constituíra as bases da política externa dos Estados Unidos para a América Latina. Então, seguindo esse importante rastro, o ponto alto da reflexão se dá quando, no último capítulo e nas considerações finais, aponta que sociologia rural comunitarista e antitotalitarismo associaram-se em defesa do centro democrático. Planejaram para o mundo rural dos dois países intervenções reformistas de terceira via: nem à esquerda, nem à direita.

A originalidade de *Em busca da comunidade* (Lopes, 2020) está na profundidade da reflexão que alcança posições políticas assumidas por sociólogos rurais ao longo da Guerra Fria. Não é um estudo do pensamento de José Arthur Rios, mas da internacionalização de ideias de democratização da vida rural dos Estados Unidos ao Brasil.

O esforço de mostrar que Rios não foi aprendiz passivo de Smith e importador de ideias americanas de democratização rural, entretanto, a meu ver, não tem rendimentos significativos nos momentos conclusivos do livro. Não terminamos a leitura convencidos de que as diferenças entre as propostas de Rios e a ideologia reformista da terceira via são grandes. A impressão ao final da leitura é que Lopes realçou a vida do espírito, a autenticidade do pensamento de Rios na tentativa de salvá-lo de julgamentos do presente. Essa impressão aumenta quando notamos certa contradição no livro. Não fica clara a razão da escolha do autor de abordar a vida e a obra do sociólogo brasileiro nos dois últimos capítulos do livro. Lopes argumenta que Rios foi um dos principais elos do Brasil com a rede internacional do comunitarismo. Essa é a razão de ele fazer parte do estudo? Seria porque Rios foi aquele que melhor expressou as ideias do comunitarismo, do reformismo, do desenvolvimento rural no Brasil de forma compatível com o modelo democrático dos Estados Unidos? Tal é a possível chave de leitura quanto à presença de Rios que se insinua ao final da leitura, mas não sem certas dubiedades em relação ao peso dos Estados Unidos e de seus cânones sociológicos nas propostas de Rios (Froehlich, 1994; Parmar, 2012).

É preciso todavia fazer justiça ao livro, esclarecendo que não vejo outras dubiedades ou ambiguidades problemáticas. A verdade é que o texto de Thiago corre com fluidez em vários momentos da leitura. É marcado pelo estilo polido e elegante de escrita da história, embora – verdade também seja dita – em certos momentos exagere nesse estilo e se aproxime de algo meio rococó; o quase rococó aparecendo aqui e ali, no modo de dizer o que se quer dizer sem dizê-lo com certeza, no uso vez ou outra do preciosismo e de palavras hoje praticamente em desuso e no rocambolesco de informações repetidas ao longo do texto sem a menor necessidade. Mas, que fique claro ao leitor, essas observações podem tranquilamente ser encaradas como pormenores pouco significativos quando olhamos para o conjunto do livro.[1]

*Em busca da comunidade* é um livro original, rico em análises fundamentadas que contribuem para a ampliação do conhecimento sobre a questão agrária. Ressalto a qualidade da investigação arqueológica da sociologia rural e suas teses reformistas. Diante de tamanha qualidade, pode-se dizer que nesse livro mora o segredo, por ele agora revelado, de tudo aquilo que estava por trás da utopia comunitária.

## NOTA

[1] Todas as vezes que vejo a capa do livro sinto desconforto e penso: essa capa não valoriza o conteúdo. Primeiro, não entendo a relação das imagens da capa com os temas desenvolvidos pelo autor. Acho importante dizer que o livro merecia uma capa própria, que expressasse sua grandeza particular, em vez de seguir o padrão editorial da Coleção História e Saúde.


REFERÊNCIAS

BLOCH Marc Apologia da história, ou, o ofício do historiador Rio de Janeiro Jorge Zahar 2001
FOUCAULT Michel A arqueologia do saber Rio de Janeiro Forense Universitária 2008
FROEHLICH José Marcos A crítica da sociologia rural “tradicional” e a busca de abordagens contemporâneas para o espaço agrário Extensão Rural 2 2 33 48 1994
LOPES Thiago da Costa Em busca da comunidade: ciências sociais, desenvolvimento rural e diplomacia cultural nas relações Brasil-EUA (1930-1950) Rio de Janeiro Editora Fiocruz 2020
MAIA João Marcelo Ehlert História da sociologia como campo de pesquisa e algumas tendências recentes do pensamento social brasileiro História, Ciências, Saúde – Manguinhos 24 1 111 128 2017 28380167 10.1590/S0104-59702017000100003
PARMAR Inderjeet Foundations of the american century: The Ford, Carnegie, and Rockefeller Foundations in the rise of American power New York Columbia University Press 2012
